# High-internal-phase emulsions stabilized by metal-organic frameworks and derivation of ultralight metal-organic aerogels

**DOI:** 10.1038/srep21401

**Published:** 2016-02-19

**Authors:** Bingxing Zhang, Jianling Zhang, Chengcheng Liu, Li Peng, Xinxin Sang, Buxing Han, Xue Ma, Tian Luo, Xiuniang Tan, Guanying Yang

**Affiliations:** 1 Beijing National Laboratory for Molecular Sciences, CAS Key Laboratory of Colloid and Interface and Thermodynamics, Institute of Chemistry, Chinese Academy of Sciences, Zhongguancun North First Street 2, Beijing 100190, China

## Abstract

To design high-internal-phase emulsion (HIPE) systems is of great interest from the viewpoints of both fundamental researches and practical applications. Here we demonstrate for the first time the utilization of metal-organic framework (MOF) for HIPE formation. By stirring the mixture of water, oil and MOF at room temperature, the HIPE stabilized by the assembly of MOF nanocrystals at oil-water interface could be formed. The MOF-stabilized HIPE provides a novel route to produce highly porous metal-organic aerogel (MOA) monolith. After removing the liquids from the MOF-stabilized HIPE, the ultralight MOA with density as low as 0.01 g·cm^−3^ was obtained. The HIPE approach for MOA formation has unique advantages and is versatile in producing different kinds of ultralight MOAs with tunable porosities and structures.

Metal-organic frameworks (MOFs) are crystalline materials that are composed of metal ions or clusters bridged by organic ligands^1^,^2^,^3^. Recently, numerous efforts have been devoted to the design of MOFs with macro- or mesoporous three-dimensional (3D) architectures due to their wide applications in different fields such as gas storage^4^,^5^,^6^,^7^, gas separation^8^,^9^,^10^, chemical sensing^11^,^12^,^13^,^14^ and catalysis^15^,^16^,^17^,^18^,^19^. Among the diverse kinds of MOF architectures, the metal-organic aerogel (MOA) has emerged as a new porous material, which is expected not only to preserve the intrinsic pore volume of MOF in a integral material but also to permit the efficient practical applications due to their easy-handling shape^20^,^21^,^22^,^23^. The MOA is generally derived from a metal-organic gel (MOG) matrix^20^,^21^,^22^,^23^. Nevertheless, the formation of MOG driven by metal-ligand coordination is often difficult because precipitation or crystallization occurs frequently^24^.

High-internal-phase emulsion (HIPE), which is also known as concentrated emulsion or gel emulsion, is emulsion that its internal phase has a high volume fraction. Because the high-content internal phase is tightly packed by the continuous phase which forms persistent microscopic liquid films, HIPE is highly viscous. Up to now, HIPEs have found wide applications in polymerization^25^,^26^, material synthesis^27^,^28^, etc. In general, surfactants are used to emulsify the immiscible solvents (water and oil) for HIPE formation^25^,^26^,^27^,^28^,^29^. Recently, there have been increasing interests in utilizing solid particles as emulsifiers for the preparation of HIPEs^30^,^31^,^32^. The particles for stabilizing HIPE are organic or inorganic particles and the functionalization for the solid particles is usually needed to modify the amphiphilicity of particles (e.g. functionalized silica and surface-active polymers particles). Moreover, the additional additives (e.g. salt, aicid, etc) and high-energy input (e.g. high shear force or high speed homogenization) are requisite for producing HIPE. To design simple HIPE systems stabilized by solid particles are of great interest, but still remain challenging.

Owing to the hybrid composition, mid-range zeta potentials and tunable structures, MOF crystallites can assembly at liquid-liquid interface^33^,^34^. Here we demonstrate for the first time the utilization of MOF for HIPE formation. It was found that the HIPE could be prepared by stirring the mixture of water, oil and MOF at room temperature, involving no additional additives or high-energy input. The MOF nanocrystals assembled at oil-water interface play an exceptional role in stabilising the HIPE. Such a MOF-stabilized HIPE with high viscosity and large internal phase volume fraction (>0.7) makes it possible to produce highly porous MOA monolith. A strategy for deriving MOA from the MOF-stabilized HIPE is proposed, as illustrated in Fig. 1. The HIPE stabilized by the assembly of pre-formed MOF nanoparticles at oil-water interface is first prepared. Then the inner oil phase and outer water phase are subsequently removed by supercritical CO_2_ replacement and freeze drying. The MOA that preserves the skeleton replica of HIPE can be thus obtained. This approach for MOA formation has unique advantages. Since the HIPE contains only three components, i.e. solid MOF and two liquids (water and oil), pure MOA solid can be easily obtained by removing the liquids and the complicated post-processing (such as washing and separating) suffering from the conventional HIPE is avoided. The route is versatile in producing different kinds of ultralight MOAs with tunable porosities and structures.

## Results

### Diethyl ether-in-water HIPE stabilized by Cu_3_(BTC)_2_

Cu_3_(BTC)_2_ (BTC = 1,3,5-benzenetricarboxylate) is one of the most widely studied MOFs and has diverse applications in different fields^35^,^36^,^37^,^38^,^39^,^40^. Here Cu_3_(BTC)_2_^41^ was used as a stabilizer for HIPE. The concentration of Cu_3_(BTC)_2_ was fixed at 10 mg·mL^−1^ of the overall volume of water and diethyl ether. For a typical synthesis, the mixture of water, diethyl ether and Cu_3_(BTC)_2_ was stirred 3 hrs at room temperature for emulsification. Then the stir was stopped and the emulsion completely separated into two phases in 8 hrs. As the diethyl ether volume fraction was in the range of 0.29~0.57, the emulsion separated into a lower phase of excess water and an upper emulsion (Fig. 2a–c). Interestingly, the upper emulsion presented gel-like or semi-solid appearance, which is a key character for the HIPE formation. The composition of the upper HIPE was calculated by subtracting the lower water. The diethyl ether volume fractions of the three HIPEs, formed from the emulsions with the initial diethyl ether volume fractions of 0.57, 0.43 and 0.29, are 0.78, 0.75 and 0.73, while the MOF concentrations are 1.5 wt%, 2.0 wt% and 2.8 wt%, respectively. Outside the diethyl ether volume fraction range of 0.29~0.57, the gel emulsion cannot form (Supplementary Fig. 1).

The microstructures of the HIPEs were characterized by confocal laser scanning microscopy (CLSM). To identify each phase in the HIPE, the water phase was marked by Rhodamine B. The droplets are polyhedral and squeeze among each other, which are bridged by Cu_3_(BTC)_2_ nanoparticles (Fig. 2d–f). This morphology is characteristic of HIPE formation, being the best geometric conformation to achieve the most dense and optimized close-packed structure^42^,^43^,^44^,^45^. The colorized CLSM images reveal that the HIPE is diethyl ether-in-water type. Namely, diethyl ether makes up the droplet, while water is the continuous phase. The average droplet sizes are 18 μm, 8 μm and 5 μm for the three HIPEs shown in Fig. 2d–f, respectively. Evidently, the droplet size decreases with the decreasing initial diethyl ether volume fraction of the emulsion. It is worth noting that the HIPE stabilized by Cu_3_(BTC)_2_ is very stable and can keep stability more than one month, which was confirmed by direct observation and CLSM images (Supplementary Fig. 2).

### Cu_3_(BTC)_2_ MOAs derived from HIPEs

The very high viscosity and large internal phase of the above HIPEs at low MOF concentration (<3.0 wt%) endow them unique advantage for forming intact and highly porous MOA monoliths. Solvent removal is a key step to preserve the porous structure and the monolith shape. The diethyl ether inside the droplet was firstly replaced by supercritical CO_2_ to keep the monolithic shape of original HIPE. CO_2_ was then released under frozen state and water was removed by lyophilization. The as-synhtesized MOA presents an aerogel appearance with an outstanding volume expansion (Fig. 3b) compared with the pristine Cu_3_(BTC)_2_ (Fig. 3a). The density of the MOA was determined to be 0.015 g·cm^−3^. Figure 3c shows that the X-ray diffraction (XRD) patterns of the pristine Cu_3_(BTC)_2_ and the MOAs synthesized from different HIPEs are nearly identical. It indicates that the MOAs preserve the crystal form of the pristine Cu_3_(BTC)_2_. No obvious difference was observed for the FT-IR spectra of the MOAs and the pristine Cu_3_(BTC)_2_ (Supplementary Fig. 3).

The scanning electron microscopy (SEM) images of the MOAs show the formation of macroporous structure (Fig. 3e–g), completely different from the morphologies of the pristine Cu_3_(BTC)_2_ (Fig. 3d). By combination with Fig. 2d–f, it can be seen that the MOAs well duplicate the respective microstructures of the HIPEs. From Fig. 3e–g, the macropore size of the MOA derived from HIPEs decreases with the decreasing initial diethyl ether volume fraction of the emulsion, in accordance with the decreased size of microdroplets in the HIPEs with different diethyl ether content (Fig. 2d–f). From the magnified SEM images shown in the inset of Fig. 3d, the pristine Cu_3_(BTC)_2_ presents particle aggregates in range of 20~80 nm. However, the nanoparticles are interconnected for the MOAs to form nanopores in dozens of nanometers (insets of Fig. 3e–g). The resutls prove that the pre-formed Cu_3_(BTC)_2_ nanoparticles can be reconstructured by HIPE.

### Porosity and mechanical properties of Cu_3_(BTC)_2_ MOAs

The macroporosities of the MOAs were determined by mercury porosimetry method after the sample was dried and degassed at 80 ^o^C. As listed in Table 1, both the total macropore volume and porosity of the MOAs greatly increase compared with those of the pristine Cu_3_(BTC)_2_. For example, the total macropore volume of the MOA synthesized in HIPE with initial diethyl ether volume fraction of 0.57 can reach 9.0 cm^3^·g^−1^, much higher than that of the pristine MOF (2.0 cm^3^·g^−1^). The porosity degrees of the three MOAs are higher than 87%. Interestingly, the degree of porosity of the MOA is higher than the volume fraction of diethyl ether in the HIPE (~75 vol% internal phase). The excess porosity higher than the volume fraction of diethyl ether can be attributed to the nanopores formed by the interconnected Cu_3_(BTC)_2_ particles, as shown in the insets of Fig. 3e–g. Moreover, it can be seen from Table 1 that the total macropore volumes and porosities of the three MOAs decrease with the decreasing initial diethyl ether volume fraction, accompanied with the increased density. It can be attributed to the decreased diethyl ether volume in the HIPE, because the diethyl ether droplets work as the templates for macropore formation. The macropore size distribution curves of the three MOAs are shown in Supplementary Fig. 4. All these curves show a bimodal size distribution, i.e. the macropores in microns with a high polydispersity and the nanopores smaller than 100 nm. As listed in Table 1, both the sizes of macropores and nanopores decrease with the decreasing initial diethyl ether volume fraction, which is consistent with that observed from the SEM images. The BET (Brunauer, Emmett and Teller) surface areas (S_BET_) of the MOAs are higher than that of the pristine Cu_3_(BTC)_2_ and increase with the decreased density (Table 1, Supplementary Fig. 5). The above results prove that the MOAs have hierarchically macro- and nanoporous architectures and their porosities can be easily modulated.

The mechanical properties of the Cu_3_(BTC)_2_ MOA monoliths were measured by uniaxial compression (see photographs of compression test and stress-strain curves in Supplementary Fig. 6). The Young’s modulus of the MOA is enhanced from 16 KPa to 34 KPa with the increased density (Table 1). In comparison with the Cu_3_(BTC)_2_ monolith prepared by powder-packing synthesis (with a Young’s modulus of 442 KPa)^46^ and the polyacrylamide-supported Cu_3_(BTC)_2_ monolith (stress values ~1.5 MPa at catastrophic failure)^47^, the Young’s modulus and compressive strength of the as-synthesized Cu_3_(BTC)_2_ MOAs are lower. It can be attributed to the extremely low densities of the as-synthesized MOAs and the absence of a secondary phase as support for MOF skeleton.

### Adsorption performance of Cu_3_(BTC)_2_ MOA

The hierarchical macro- and nanoporous 3D architecture of the as-synthesized MOA would promote the diffusion of guest molecules into the pores (and active sites) of the framework. Here the elimination of different dyes in aqueous solutions by Cu_3_(BTC)_2_ MOA was tested. The MOA shows enhanced adsorption capability for methylene blue (MB) and Rhodamine B (RHB) than the pristine Cu_3_(BTC)_2_ (Fig. 4a). 86% of MB can be adsorbed by MOA within 1.5 min, while only 48% of MB can be adsorbed by the pristine Cu_3_(BTC)_2_. Upon adsorption equilibrium, the adsorption capacity (the adsorption amount of the dye per gram of the absorbent) of MOA can reach 94.3 mg·g^−1^, higher than that of the pristine Cu_3_(BTC)_2_ (78.2 mg·g^−1^). Also, the time taken to reach equilibrium is shortened by MOA (Fig. 4a). The adsorption capacity of the as-synthesized MOA is much higher than that of the reported Cr-BTC MOA over 1000 min at the same condition (20.2 mg·g^−1^)^48^. The Cu_3_(BTC)_2_ could be negatively charged under neutral condition and adsorb the positively charged dye cations through electrostatic attractions^49^. Due to the highly porous structure of the Cu_3_(BTC)_2_ MOA interconnected by nanoparticles, the transport limitation can be efficiently reduced, which is favorable for the accessibility to the active site of cationic charge. Therefore, the Cu_3_(BTC)_2_ MOA shows an enhanced adsorption capacity for MB molecules in aqueous solution. Further, the performance of the as-synthesized MOA for CO_2_ capture was studied. At 1 atm, the CO_2_ uptake for MOA-1 is 135 cm^3^·g^−1^ (Fig. 4b), which is a 17% increase than the pristine Cu_3_(BTC)_2_. In sharp contrast, the CO_2_ uptake of the MOAs obtained from the gelation in pure ethanol at the same condition is less than 50 cm^3^·g^−1^ 21. From Fig. 4b, the MOA shows much lower adsorption for CH_4_ and N_2_ than CO_2_. It indicates that the MOA can be used as a promising candidate for the separation of CO_2_/N_2_ and CO_2_/CH_4_ with a high selectivity.

### Cyclohexane-in-water HIPEs stabilized by Cu_3_(BTC)_2_

**Figure 1:**
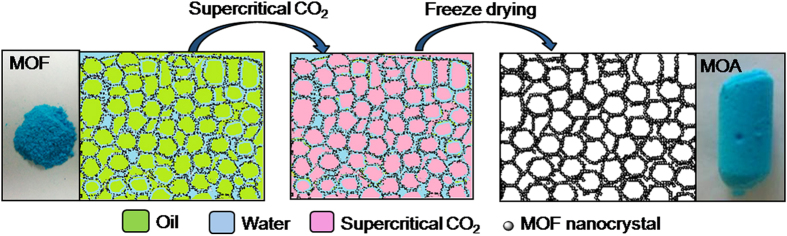
Diagram illustrating the MOF-stabilized HIPE and derivation of MOA from HIPE.

**Figure 2:**
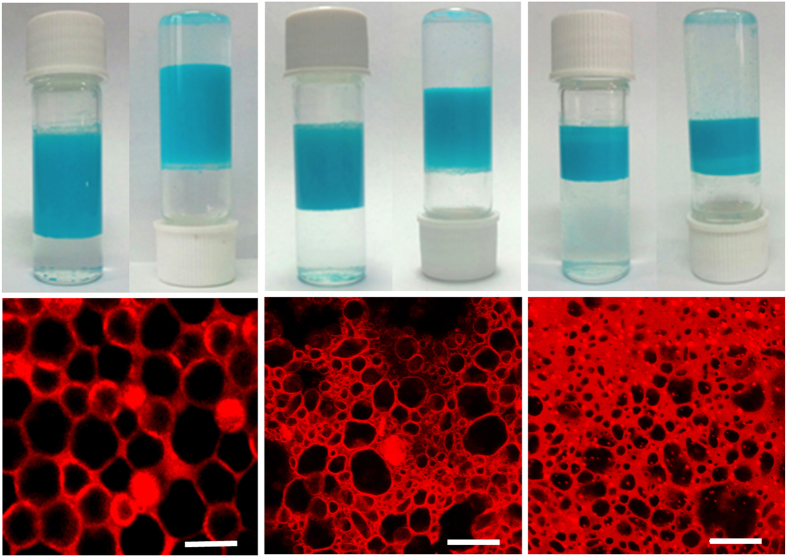
Characterization of Cu_3_(BTC)_2_-stabilized HIPEs. (**a-c**) Photographs of the emulsions stabilized by Cu_3_(BTC)_2_ with the initial diethyl ether volume fractions of 0.57, 0.43 and 0.29, respectively. (**d-f**) The corresponding CLSM images of the above HIPEs (HIPE-1, HIPE-2 and HIPE-3, respectively). Scale bars, 20 μm.

**Figure 3:**
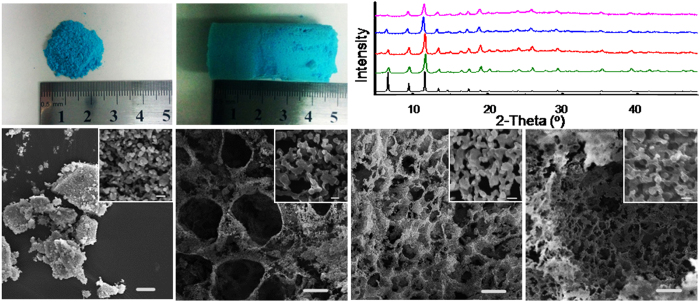
Characterization of the pristine Cu_3_(BTC)_2_ and MOAs. (**a,b**) Photographs of the pristine Cu_3_(BTC)_2_ and MOA-1 synthesized from HIPE-1; (**c**) Simulated XRD pattern of Cu_3_(BTC)_2_ (black), XRD patterns of MOF (green), MOA-1 (red), MOA-2 (blue) and MOA-3 (pink) synthesized from HIPE-1, HIPE-2 and HIPE-3, respectively; (**d–g**) SEM images of the pristine Cu_3_(BTC)_2_, MOA-1, MOA-2 and MOA-3. Scale bars, 10 μm in images (**d–g**) and 100 nm in the insets.

**Figure 4:**
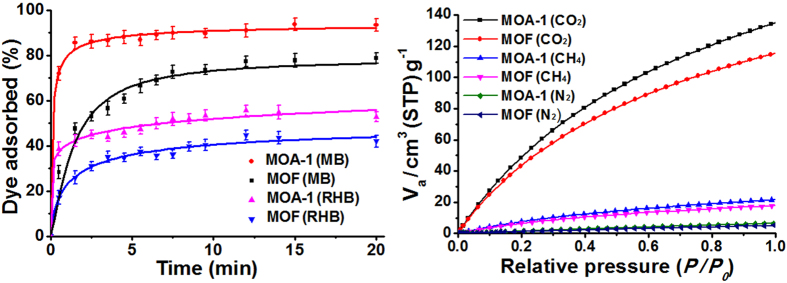
Adsorption performance of the pristine Cu_3_(BTC)_2_ and MOA. (**a**) Kinetic adsorption curves of 10 mg MOA-1 (or the pristine Cu_3_(BTC)_2_) in 10 mL of MB or RHB solutions (100 mg·L^−1^), respectively. (**b**) CO_2_, CH_4_ and N_2_ adsorption isotherms of the pristine Cu_3_(BTC)_2_ and MOA-1 at 273.2 K.

Cyclohexane was used as an alternative of diethyl ether to form MOF-stabilized HIPE with water. The results show that the cyclohexane-in-water HIPEs stabilized by Cu_3_(BTC)_2_ nanoparticles can be prepared (see photograph and CLSM image in Supplementary Fig. 7), similar to those of the diethyl ether-in-water HIPEs shown in Figs 1 and 2. The MOA monoliths were derived from the HIPEs after removing cyclohexane and water (Supplementary Fig. 7). The densities of the Cu_3_(BTC)_2_ MOAs obtained from HIPEs with the initial cyclohexane volume fractions of 0.57, 0.43 and 0.29 are 0.0096 g·cm^−3^, 0.014 g·cm^−3^ and 0.028 g·cm^−3^, respectively. The MOAs well preserve the crystal form of the pristine Cu_3_(BTC)_2_ (see XRD patterns in Supplementary Fig. 7).

### HIPEs stabilized by Mn_3_(BTC)_2_ and Ni(BDC)

**Figure 5:**
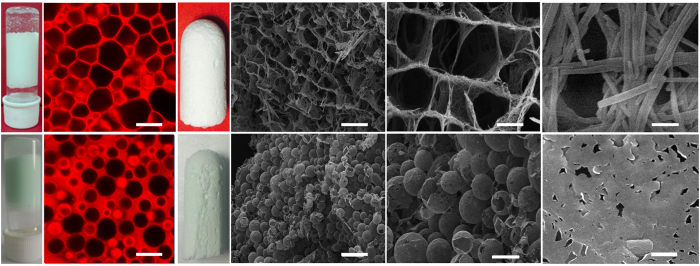
Characterization of the HIPEs stabilized by Mn_3_(BTC)_2_ and Ni(BDC) and the corresponding MOAs. (**a,b**) Photograph and CLSM image of the HIPE stabilized by Mn_3_(BTC)_2_; (**c–f)** Photograph and SEM images of Mn_3_(BTC)_2_ MOA. (**g,h**) Photograph and CLSM image of the HIPE stabilized by Ni(BDC); (**i–l**) Photograph and SEM images of Ni(BDC) MOA. Scale bars, 20 μm (**b,d,h,k**), 10 μm (**e**), 50 μm (**j**) and 500 nm (**f,l**).

The possibility of stabilizing HIPE by other MOFs was investigated. First, a one-dimensional (1D) MOF, i.e. Mn_3_(BTC)_2_ nanowire, was synthesized (Supplementary Fig. 8) and used for the emulsification of water and cyclohexane (1:1 in volume ratio). The gel-like HIPE with polyhedral cyclohexane droplets was formed (Fig. 5a,b). The cyclohexane volume fraction of the HIPE was determined to be 0.78. After extracting the solvents from HIPE, the ultralight Mn_3_(BTC)_2_ MOA with a density of 0.014 g·cm^−3^ was obtained (Fig. 5c). The Mn_3_(BTC)_2_ MOA presents a morphology of honeycomb structure (Fig. 5d,e). The pore shape and size are similar to those of the polyhedral droplets shown in Fig. 5b. It confirms that the Mn_3_(BTC)_2_ MOA can well preserve the structure of HIPE. From the magnified SEM image shown in Fig. 5f, it is evident that the wall of the macropore is stacked by MOF nanowires. The diameter and length of the nanowires are similar to those of the pristine Mn_3_(BTC)_2_ nanowires (Supplementary Fig. 8). The results indicate that the HIPE can conduct the reconstruction of the pre-formed MOF nanocrystals, while it has no influence on the shape and size of the MOF unit.

Further, the two-dimensional (2D) Ni(BDC) (BDC = 1,4-benzenedicarboxylate) nanosheets were synthesized (Supplementary Fig. 9) for HIPE formation. The 2D Ni(BDC) also produced the gel-like HIPE in 1:1 cyclohexane/water mixture (Fig. 5g). However, the droplets are spherical and less crowded than those in the HIPEs stabilized by Cu_3_(BTC)_2_ and Mn_3_(BTC)_2_ (Fig. 5h). It can be partly due to the lower cyclohexane volume fraction (0.73) of the HIPE stabilized by Ni(BDC). Besides, due to steric repulsion effect, the 2D structure of Ni(BDC) nanosheet may be unfavorable for the efficient linkage of MOF crystals to form a HIPE structure as compact as the above two HIPEs stabilized by 0D and 1D nanocrystals. The Ni(BDC) MOA obtained from the HIPE (Fig. 5i) has a density of 0.012 g·cm^−3^. It presents a morphology of hollow spheres (Fig. 5j,k) and their sizes distribute in a similar range with the HIPE droplets shown in Fig. 5h (5–20 μm). As can be seen from the magnified SEM image shown in Fig. 5l, the wall of the hollow sphere is ultrathin and formed by the connection of nanosheets along the surface. The Mn_3_(BTC)_2_ and Ni(BDC) MOAs well preserve the respective crystal forms of the pristine MOFs (Supplementary Figs 10 and 11). The above results prove that the HIPEs can be stabilized by different MOFs, which are versatile in producing MOAs with modulated structures.

### Mechanism

**Figure 6:**
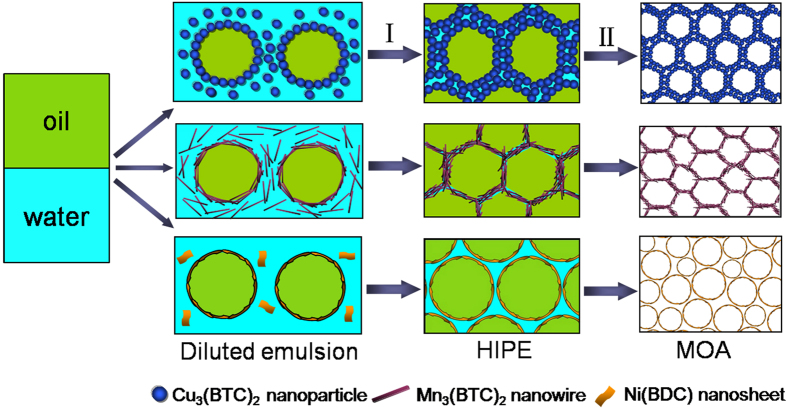
Diagram illustrating the process for the formation of HIPEs stabilized by different MOF nanocrystals and the derivation of MOAs. I, Separating excess water from diluted emulsion; II, removing solvents from HIPE.

Zeta potential is usually used to judge the hydrophilic property of particles and the electrostatic interaction among particles^50^. To explore the underlying mechanism for the HIPE formation, the zeta potentials of the MOFs in water were determined. The zeta potentials of Cu_3_(BTC)_2_, Mn_3_(BTC)_2_ and Ni(BDC) in water (1 mg·mL^−1^) were –0.54 mV, –2.42 mV and –0.42 mV, respectively. All the three MOFs have negative and mid-range zeta potentials, allowing suitable amphiphilicity and weak repulsions between MOF nanocrystals. Therefore, the MOF nanocrystals can be anchored at oil-water interface and emulsify the two immiscible phases (Fig. 6a). In addition to the nanocrystals adsorbed at oil-water interface, there are nanocrystals that dispersed in aqueous phase.

The diluted emulsion with a large amount of water was unstable, resulting in water sedimentation and creaming up of oil drops^34^,^51^. During this process, the nanocrystals adsorbed at oil-water interface are probably contiguous with those dispersed in aqueous phase. Among the three MOFs, Mn_3_(BTC)_2_ nanocrystals have the weakest electrostatic repulsion between nanocrystals in continuous phase and interfacial nanocrystals. So the Mn_3_(BTC)_2_ nanocrystals from different oil droplets and continuous phase are bound together into a 3D network, which in turn traps the oil droplets in gel matrix (Fig. 6b). Cu_3_(BTC)_2_ and Ni(BDC) have similar zeta potentials; however, the 2D nanosheet structure of Ni(BDC) creates a steric barrier for the effective linkage of nanocrystals into a network. Consequently, Ni(BDC) nanosheets are adsorbed at oil-water interface to form spherical droplets (Fig. 6b). The gel-like HIPE was much stable because the continuous phase filled with nanocrystals was highly viscous and the nanocrystals at oil-water interface can cross link into network or aggregate closely to hinder droplet coalescence^50^ (Fig. 6b). After extracting the liquids, the HIPEs stabilized by Cu_3_(BTC)_2_ and Mn_3_(BTC)_2_ produced 3D interconnected MOAs, while the HIPE stabilized by Ni(BDC) favored the formation of MOA with spherical capsules (Fig. 6c).

## Discussion

Here the formation of a MOF-stabilized HIPE was proposed. It was found that the MOF nanocrystals, including 0D nanoparticles, 1D nanowires and 2D nanosheets, could emulsify the two immiscible solvents (water and oil) and produce stable HIPEs. The structures of HIPEs can be easily tuned by adjusting the properties of MOF nanocrystals and oil-to-water ratio. The MOF nanocrystals assembled at oil-water interface play an exceptional role in stabilising the HIPE. In comparison with the conventional HIPEs, the MOF-stabilized HIPE has many advantages. For example, the HIPE could be prepared by stirring the mixture of water, oil and MOF at room temperature, involving no additional additives or high-energy input. Also, the functionalization for the commonly used organic or inorganic particles for HIPE formation is avoided due to the hybrid and amphiphilic property of MOF itself.

The above HIPEs with high viscosity and low MOF concentration (<3.0 wt%) endow them unique merits in producing intact and highly porous MOA monoliths. The ultralight MOA monoliths with densities as low as 0.01 g·cm^−3^ were obtained after extracting the liquids, which well preserve the skeleton replicas of HIPEs. It is worth noting that MOA is generally derived from a MOG matrix^20^,^21^,^22^,^23^,^24^, which is often difficult due to the particle precipitation or crystallization during the gelation process. To get the desired MOG, heating for the solution of precursors (>80 ^o^C) and aging for the wet gel up to days are usually requisite. The MOF-stabilized HIPE route is facile and can be applied to the synthesis of different kinds of MOA monoliths. We anticipate that more MOAs and MOA composites (e.g. MOA/MOA, metal/MOA, metal oxide/MOA, etc) with unique features would be prepared by the HIPE route and find applications in catalysis, adsorption and energy storage.

## Methods

### MOF synthesis

Cu_3_(BTC)_2_ was synthesized according to literature^41^. Typically, Cu(OAc)_2_·H_2_O (2.0 g) and H_3_BTC (1.4 g) were respectively dissolved in a 1:1 water/ethanol mixture (25 mL). Then the two solutions were mixed and stirred rapidly for 1 h. The precipitate was centrifugated, washed with ethanol and dried under vacuum. For the synthesis of Mn_3_(BTC)_2_, MnCl_2_ (1.89 g) and H_3_BTC (2.1 g) were mixed with 50 mL ethanol under vigorous agitation. 2 mL triethylamine was added into the above solution at room temperature. After reaction for 6 hrs, the white precipitate was washed with ethanol and dried at 60 ^o^C under vacuum for 24 hrs. For the synthesis of Ni(BDC), NiCl_2_·6H_2_O (1.5 g) and H_2_BDC (2.0 g) were added into ethanol (50 mL), the other experimental procedures and conditions being the same as those for the Mn_3_(BTC)_2_ synthesis.

### Emulsion formation and characterization

The desired amount of MOF was added into the water-oil mixture with a certain oil volume fraction and the mixture was stirred at room temperature for 3 hrs. The MOF concentration was fixed at 10 mg·mL^−1^ of the overall emulsion volume. Then the mixture was kept without agitation to allow phase separation. The lower water was removed by injector and the upper HIPE was characterized by OLYMPUS FV1000-IX81 confocal laser scanning microscopy with excitation wavelength of 559 nm. 5.0 μL emulsion containing Rhodamine B (10^−4^ M) was trickled on a 0.7 mm thick cover slip through microsyringe and covered with another, and was monitored and captured by a digital CCD.

### MOA formation and characterization

The as-prepared HIPE was first treated by supercritical CO_2_ to remove oil and then frozen at –20 ^o^C for 2 hrs. CO_2_ was released slowly and the sample was lyophilized for 20 hrs to remove water. Then the product was obtained. The morphologies of the MOAs were characterized by scanning electron microscope (SEM, HITACHI S-4800). XRD was performed on a Rigaku D/max-2500 diffractometer with Cu Kα radiation (λ = 1.5418 Å) at 40 kV and 200 mA. The macroporosities were recorded by mercury intrusion porosimetry using a Micromeritics Autopore IV 9500 porosimeter. The sample was subjected to a pressure cycle starting at 5 psia, increasing to 44500 psia in predefined steps to give pore size/pore volume information. N_2_ adsorption-desorption isotherms were obtained using a Micromeritics ASAP 2020M system. The FT-IR spectra were obtained using a Bruker Tensor 27 spectrometer. Malvern Zetasizer Nano-ZS instrument (ZEN3600, Malvern Instruments, Worcestershire, UK), equipped with a 4 mW He-Ne laser (wavelength 633 nm), was used to determine zeta potential of the MOF mixed with water. The concentration of MOF suspension was fixed at 1 mg·mL^−1^ and the measurement was conducted at 25.0 ± 0.1 ^o^C. The compression test was carried out using a model 3342 Instron Universal Testing Machine at a rate of 20% strain min^−1^.

### Adsorption performance of MOA

The test was carried out by continuously shaking the mixture of 10 mL dye aqueous solution (100 mg L^−1^) with the MOA or MOF (10 mg) for different times. After centrifuging, the dye concentration was analyzed using a Shimadzu UV-2450 UV-vis spectrophotometer. For the gas adsorption test, the adsorption isotherms of CO_2_, N_2_ and CH_4_ were recorded at 273.2 K for MOAs and pristine MOF in pressure range of 0.0004–1 atm on a TriStar II 3020 device. For each measurement, about 100 mg of the sample was degassed using the same procedure.

## Additional Information

**How to cite this article**: Zhang, B. *et al.* High-internal-phase emulsions stabilized by metal-organic frameworks and derivation of ultralight metal-organic aerogels. *Sci. Rep.* **6**, 21401; doi: 10.1038/srep21401 (2016).

## Supplementary Material

Supplementary Information

## Figures and Tables

**Table 1:** Density, porosity and mechanical properties of the pristine Cu_3_(BTC)_2_ and MOAs.

MOF/MOAs	Density^a^/g·cm^−3^	Porosity^b^/%	V_pore_^b^/cm^3^·g^−1^	D_macro_^b^/μm	D_nano_^b^/nm	S_BET_^c^/m^2^·g^−1^	Young’s modulus/KPa
MOF	–	72.1	2.0	6.7	–	307	–
MOA-1	0.015	89.7	9.0	19.4	95	834	16
MOA-2	0.021	88.6	8.4	16.2	77	584	21
MOA-3	0.032	87.7	7.6	14.8	62	320	34

^a^determined by weighing method.

^b^determined by mercury porosimetry method.

^c^determined by N_2_ adsorption-desorption method.

## References

[b1] Furukawa H., Müller U. & Yaghi O. M. “Heterogeneity within Order” in metal-organic frameworks. Angew. Chem. Int. Ed. 54, 3417–3430 (2015). 10.1002/anie.201410252 25586609

[b2] Furukawa H., Cordova K. E., O’Keeffe M. & Yaghi O. M. The chemistry and applications of metal-organic frameworks. Science 341, 1230444 (2013). 23990564 10.1126/science.1230444

[b3] Burtch N. C., Jasuja H. & Walton K. S. Water Stability and Adsorption in metal-organic frameworks. Chem. Rev. 114, 10575–10612 (2014). 25264821 10.1021/cr5002589

[b4] Murray L. J., Dincă M. & Long J. R. Hydrogen storage in metal-organic frameworks. Chem. Soc. Rev. 38, 1294–1314 (2009). 19384439 10.1039/b802256a

[b5] Makal T. A., Li J.-R., Lua W. & Zhou H.-C. Methane storage in advanced porous materials. Chem. Soc. Rev. 41, 7761–7779 (2012). 22990753 10.1039/c2cs35251f

[b6] He Y., Zhou W., Qian G. & Chen B. Methane storage in metal-organic frameworks. Chem. Soc. Rev. 43, 5657–5678 (2014). 24658531 10.1039/c4cs00032c

[b7] Yan Y., Yang S., Blake A. J. & Schröder M. Studies on metal-organic frameworks of Cu(II) with isophthalate linkers for hydrogen storage. Acc. Chem. Res. 47, 296–307 (2014). 24168725 10.1021/ar400049h

[b8] Li J.-R., Kuppler R. J. & Zhou H.-C. Selective gas adsorption and separation in metal-organic frameworks. Chem. Soc. Rev. 38, 1477–1504 (2009). 19384449 10.1039/b802426j

[b9] Voorde B. V., Bueken B., Denayer J. & Vos D. D. Adsorptive separation on metal-organic frameworks in the liquid phase. Chem. Soc. Rev. 43, 5766–5788 (2014). 24647892 10.1039/c4cs00006d

[b10] Li J.-R., Sculley J. & Zhou H.-C. Metal-organic frameworks for separations. Chem. Rev. 112, 869–932 (2012). 21978134 10.1021/cr200190s

[b11] Douvali A. *et al.* Turn-on luminescence sensing and real-time detection of traces of water in organic solvents by a flexible metal-organic framework. Angew. Chem. Int. Ed. 54, 1651–1656 (2015). 10.1002/anie.201410612 25487062

[b12] Gassensmith J. J. *et al.* A Metal-organic framework-based material for electrochemical sensing of carbon dioxide. J. Am. Chem. Soc. 136, 8277–8282 (2014). 24827031 10.1021/ja5006465

[b13] Zhang M. *et al.* Two-dimensional metal-organic framework with wide channels and responsive turn-on fluorescence for the chemical sensing of volatile organic compounds. J. Am. Chem. Soc. 136, 7241–7244 (2014). 24824627 10.1021/ja502643p

[b14] Campbell M. G., Sheberla D., Liu S. F., Swager T. M. & Dinca M. Cu_3_(hexaiminotriphenylene)_2_: An electrically conductive 2D metal-organic framework for chemiresistive sensing. Angew. Chem. Int. Ed. 54, 4349–4352 (2015). 10.1002/anie.201411854 25678397

[b15] Corma A., GarcÍa, H. & Llabrés i Xamena & F. X. Engineering metal organic frameworks for heterogeneous catalysis. Chem. Rev. 110, 4606–4655 (2010). 20359232 10.1021/cr9003924

[b16] Dhakshinamoorthy A. & Garcia H. Catalysis by metal nanoparticles embedded on metal-organic frameworks. Chem. Soc. Rev. 41, 5262–5284 (2012). 22695806 10.1039/c2cs35047e

[b17] Falkowski J. M. *et al.* Privileged Phosphine-based metal-organic frameworks for broad-scope asymmetric catalysis. J. Am. Chem. Soc. 136, 5213–5216 (2014). 24684238 10.1021/ja500090y

[b18] Dhakshinamoorthy A., Asiric A. M. & Garcia H. Metal-organic frameworks catalyzed C-C and C-heteroatom coupling reactions. Chem. Soc. Rev. 44, 1922–1947 (2015). 25608717 10.1039/c4cs00254g

[b19] Manna K., Zhang T., Greene F. X. & Lin W. Bupyridine- and phenanthroline-based metal-organic frameworks for highly efficient and tandem catalytic organic transformations via directed C-H activation. J. Am. Chem. Soc. 137, 2665–2673 (2015). 25640998 10.1021/ja512478y

[b20] Lohe M. R., Rose M. & Kaskel S. Metal-organic framework (MOF) aerogels with high micro- and macroporosity. Chem. Commun. 40, 6056–6058 (2009). 10.1039/b910175f 19809642

[b21] Li L. *et al.* A synthetic route to ultralight hierarchically micro/mesoporous Al(III)-carboxylate metal-organic aerogels. Nat. Commun. 4, 1774 (2013). 23653186 10.1038/ncomms2757 PMC3644084

[b22] Xia W. *et al.* Facile and economical synthesis of metal-organic framework MIL-100(Al) gels for high efficiency removal of microcystin-LR. RSC Adv. 3, 11007–11013 (2013).

[b23] Li H. *et al.* Luminescent metal-organic gels with tetraphenylethylene moieties: Porosity and aggregation-induced emission. RSC Adv. 3, 16340–16344 (2013).

[b24] Piepenbrock M. O. M., Lloyd G. O., Clarke N. & Steed J. W. Metal- and anion-binding supramolecular gels. Chem. Rev. 110, 1960–2004 (2010). 20028020 10.1021/cr9003067

[b25] Butler R., Hopkinson I. & Cooper A. I. Synthesis of porous emulsion-templated polymers using high internal phase CO_2_-in-water emulsions. J. Am. Chem. Soc. 125, 14473–14481 (2003). 14624597 10.1021/ja037570u

[b26] Schüler F. *et al.* Synthesis of macroporous polystyrene by the polymerization of foamed emulsions. Angew. Chem. Int. Ed. 51, 2213–2217 (2012). 10.1002/anie.201107806 PMC3415665 22266818

[b27] Oschatz M. *et al.* Carbide-derived carbon monoliths with hierarchical pore architectures. Angew. Chem. Int. Ed. 51, 7577–7580 (2012). 10.1002/anie.201200024 22696469

[b28] Zhang H. F. & Cooper A. I. Synthesis and applications of emulsion-templated porous materials. Soft Matter 1, 107–113 (2005). 10.1039/b502551f 32646082

[b29] Dhanuka V. V., Dickson J. L., Ryoo W. & Johnston K. P. High internal phase CO_2_-in-water emulsions stabilized with a branched nonionic hydrocarbon surfactant. J. Colloid Interface Sci. 298, 406–418 (2006). 16376919 10.1016/j.jcis.2005.11.057

[b30] Ikem V. O., Menner A. & Bismarck A. High internal phase emulsions stabilized solely by functionalized silica particles. Angew. Chem. Int. Ed. 47, 8277–8279 (2008). 10.1002/anie.200802244 18814159

[b31] Li Z., Ming T., Wang J. & Ngai T. High internal phase emulsions stabilized solely by microgel particles. Angew. Chem. Int. Ed. 45, 8490–8493 (2009). 10.1002/anie.200902103 19798705

[b32] Sun G., Li Z. & Ngai T. Inversion of particle-stabilized emulsions to form high-internal-phase emulsions. Angew. Chem. Int. Ed. 49, 2163–2166 (2010). 10.1002/anie.200907175 20175179

[b33] Huo J., Marcello M., Garai A. & Bradshaw D. MOF-polymer composite microcapsules derived from pickering emulsions. Adv. Mater. 25, 2717–2722 (2013). 23554180 10.1002/adma.201204913

[b34] Xiao B., Yuan Q. & Williams R. A. Exceptional function of nanoporous metal organic framework particles in emulsion stabilization. Chem. Commun. 49, 8208–8210 (2013). 10.1039/c3cc43689f 23925149

[b35] Chui S. S. Y., Lo S. M. F., Charmant J. P. H., Orpen A. G. & Williams I. D. A chemically functionalizable nanoporous material [Cu_3_(TMA)_2_(H_2_O)_3_]_n_. Science 283, 1148–1150 (1999). 10024237 10.1126/science.283.5405.1148

[b36] Qiu L.-G. *et al.* Hierarchically micro- and mesoporous metal-organic frameworks with tunable porosity. Angew. Chem. Int. Ed. 47, 9487–9491 (2008). 10.1002/anie.200803640 18972472

[b37] Xiang S., Zhou W., Gallegos J. M., Liu Y. & Chen B., Exceptionally high acetylene uptake in a microporous metal-organic framework with open metal sites. J. Am. Chem. Soc. 131, 12415–12419 (2009). 19705919 10.1021/ja904782h

[b38] Sun L.-B., Li J.-R., Park J. & Zhou H.-C. Cooperative template-directed assembly of mesoporous metal-organic frameworks. J. Am. Chem. Soc. 134, 126–129 (2012). 22148588 10.1021/ja209698f

[b39] Peng L. *et al.* Surfactant-directed assembly of mesoporous metal-organic framework nanoplates in ionic liquids. Chem. Commun. 48, 8688–8690 (2012). 10.1039/c2cc34416e 22820744

[b40] Peng L. *et al.* Highly mesoporous metal-organic framework assembled in a switchable solvent. Nat. Commun. 5, 5465 (2014). 25047059 10.1038/ncomms5465 PMC4109014

[b41] Zhang B. *et al.* Solvent determines the formation and properties of metal-organic frameworks. RSC Adv. 5, 37691–37696 (2015).

[b42] Li J., Zhang J., Zhao Y., Han B. & Yang G. High-internal-ionic liquid-phase emulsions. Chem. Commun. 48, 994–996 (2012). 10.1039/c2cc15922h 22143286

[b43] Zhang D. & Clegg P. S. Relationship between high internal-phase Pickering emulsions and catastrophic inversion. Soft Matter 9, 7042–7048 (2013).

[b44] Lee K.-Y., Blaker J. J., Murakami R., Heng J. Y. Y. & Bismarck A. Phase behavior of medium and high internal phase water-in-oil emulsions stabilized solely by hydrophobized bacterial cellulose nanofibrils. Langmuir 30, 452–460 (2014). 24400918 10.1021/la4032514

[b45] Zheng X., Zhang Y., Wang H. & Du Q. Interconnected macroporous polymers synthesized from silica particle stabilized high internal phase emulsions. Macromolecules 47, 6847–6855 (2014).

[b46] Ahmed A., Forster M., Clowes R., Myers P. & Zhang H. F. Hierarchical porous metal-organic framework monoliths. Chem. Commun. 50, 14314–14316 (2014). 10.1039/c4cc06967f 25269109

[b47] Moitra N. *et al.* Mechanically stable, hierarchically porous Cu_3_(btc)_2_ (HKUST-1) monoliths via direct conversion of copper(II) hydroxide-based monoliths. Chem. Commun. 51, 3511–3514 (2015). 10.1039/c4cc09694k 25572361

[b48] Xiang S. L. *et al.* Porous organic-inorganic hybrid aerogels based on Cr^3+^/Fe^3+^ and rigid bridging carboxylates. J. Mater. Chem. A. 22, 1862–1867 (2012).

[b49] Lin S. *et al.* Adsorption behavior of metal-organic frameworks for methylene blue from aqueous solution. Micropor. Mesopor. Mater. 193, 27–34 (2014).

[b50] Tan H. *et al.* Gelatin particle-stabilized high internal phase emulsions as nutraceutical containers. ACS Appl. Mater. Interfaces 6, 13977–13984 (2014). 25102954 10.1021/am503341j

[b51] Aveyard R, Binks B. P. & Clint J. H. Emulsions stabilised solely by colloidal particles. Adv. Colloid Interface Sci. 503, 100–102 (2003).

