# Supplementary material for: High-internal-phase emulsions stabilized by metal-organic frameworks and derivation of ultralight metal-organic aerogels
Source: Sci Rep. 2016 Feb 19;6:21401. doi: 10.1038/srep21401 (PMC4759572; doi:10.1038/srep21401)
Supplement: Supplementary Information [file srep21401-s1.pdf]

High-internal-phase emulsions stabilized by metal-organic frameworks and derivation of ultralight metal-organic aerogels

Bingxing Zhang, Jianling Zhang*, Chengcheng Liu, Li Peng, Xinxin Sang, Buxing Han, Xue Ma, Tian Luo, Xiuniang Tan, Guanying Yang

1. Materials

98% $Cu(OAc)_2 \cdot H_2O$ and anhydrous $MnCl_2$ were supplied by Alfa Aesar. 99% 1,3,5-benzenetricarboxylic acid (H_3BTC), 99% 1,4-benzenedicarboxylic acid (H_2BDC), 99% triethylamine, and 99.7% cyclohexane were purchased from J&K Scientific Co., Ltd. 98% $NiCl_2 \cdot 6H_2O$ was purchased from Sinopharm Group Co., Ltd. 99% Rhodamine B and 99% Methylene Blue were purchased from Adamas Reagent Co., Ltd. Anhydrous ethanol, anhydrous ether and deionized water were provided by Beijing Chemical Works. All these materials were used directly without further purification.

2. Results

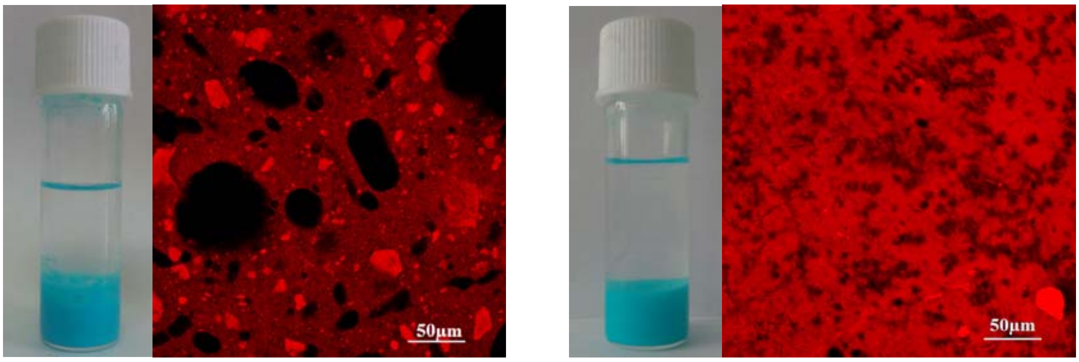

(a) (b)

Figure 1. Photographs and CLSM photographs of the emulsions stabilized by $Cu_3(BTC)_2$ with ether volume fractions of 0.86 (a) and 0.14 (b).

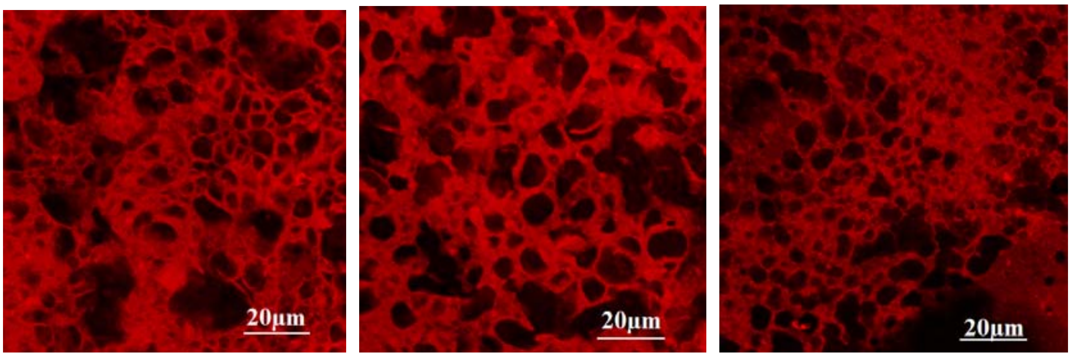

(a) (b) (c)

Figure 2. CLSM photographs of the high-internal-phase emulsions (HIPEs) stabilized by $Cu_3(BTC)_2$ with ether volume fractions of 0.57 (a), 0.43 (b), and 0.29 (c) after kept at room temperature for one month.

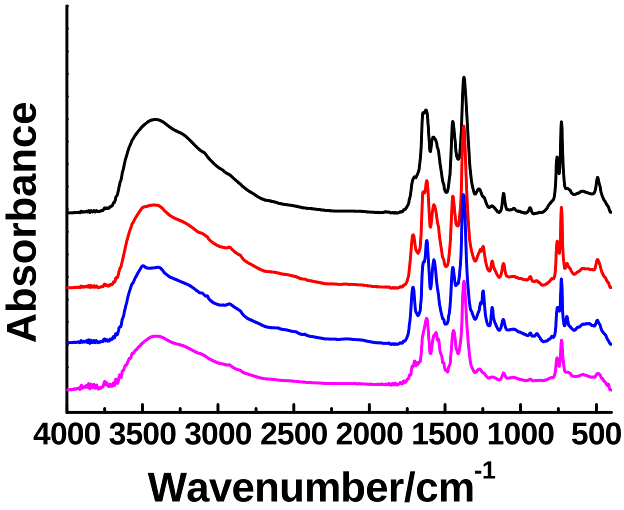

Figure 3. FT-IR spectra of the pristine $Cu_3(BTC)_2$ MOF (pink) and the MOAs synthesized from HIPEs with the ether volume fractions of 0.57 (blue), 0.43 (red), and 0.29 (black).

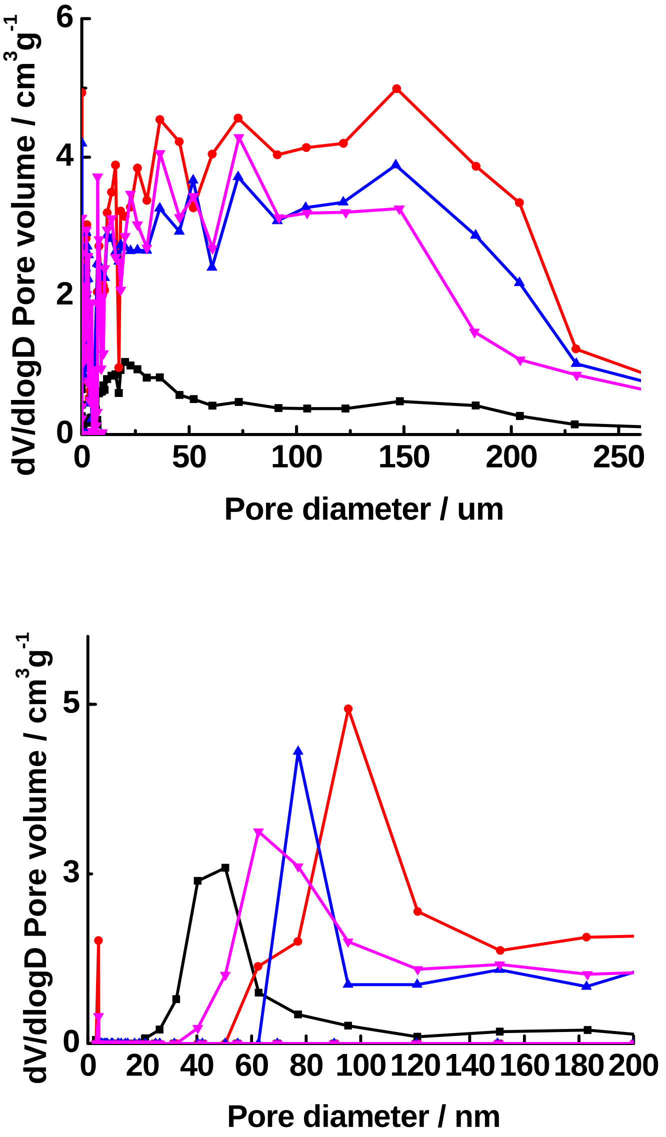

Figure 4. Macropore size distribution curves of the pristine $Cu_3(BTC)_2$ MOF (black) and the MOAs synthesized from HIPEs with the ether volume fractions of 0.57 (red), 0.43 (blue), and 0.29 (pink).

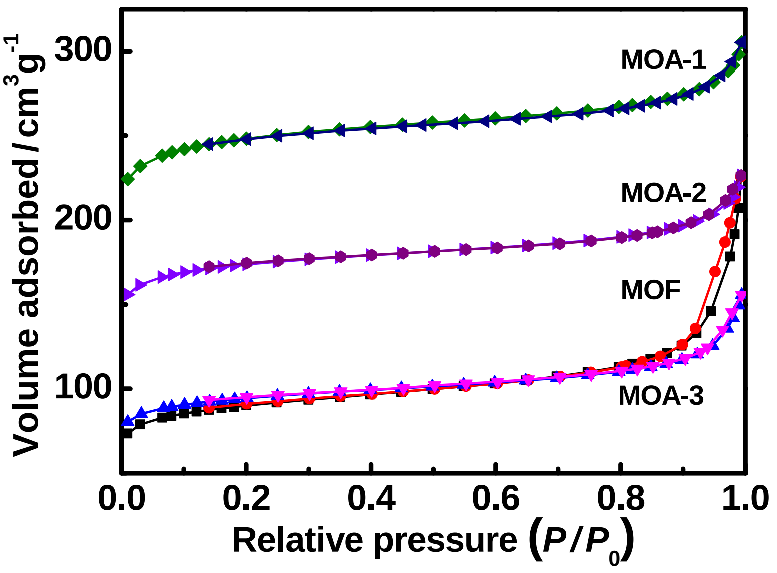

Figure 5. N_2 adsorption-desorption isotherms of the pristine $Cu_3(BTC)_2$ MOF, MOA-1, MOA-2, and MOA-3. The N_2 adsorption-desorption isotherm exhibits the mode of type I, indicative of the absence of mesopores in the MOAs.

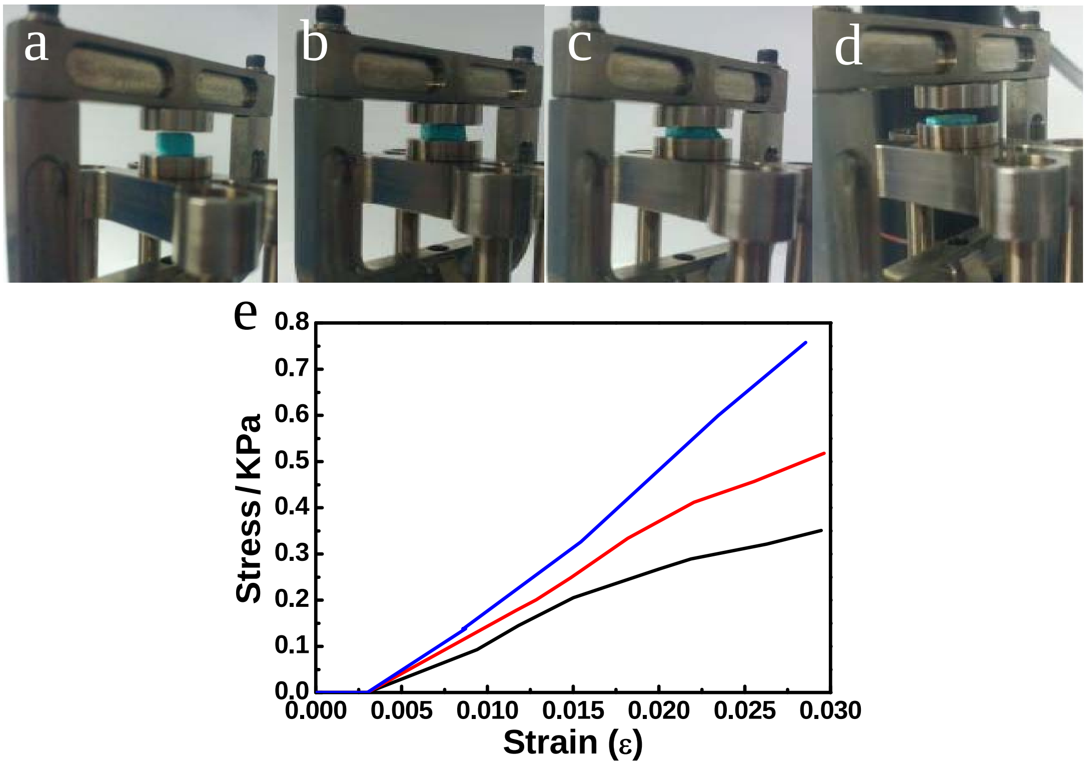

Figure 6. a-d: Photographs of MOA-1 in compression process. **e:** Stress-strain curves of a uniaxial compression test on MOA-1 (black), MOA-2 (red) and MOA-3 (blue).

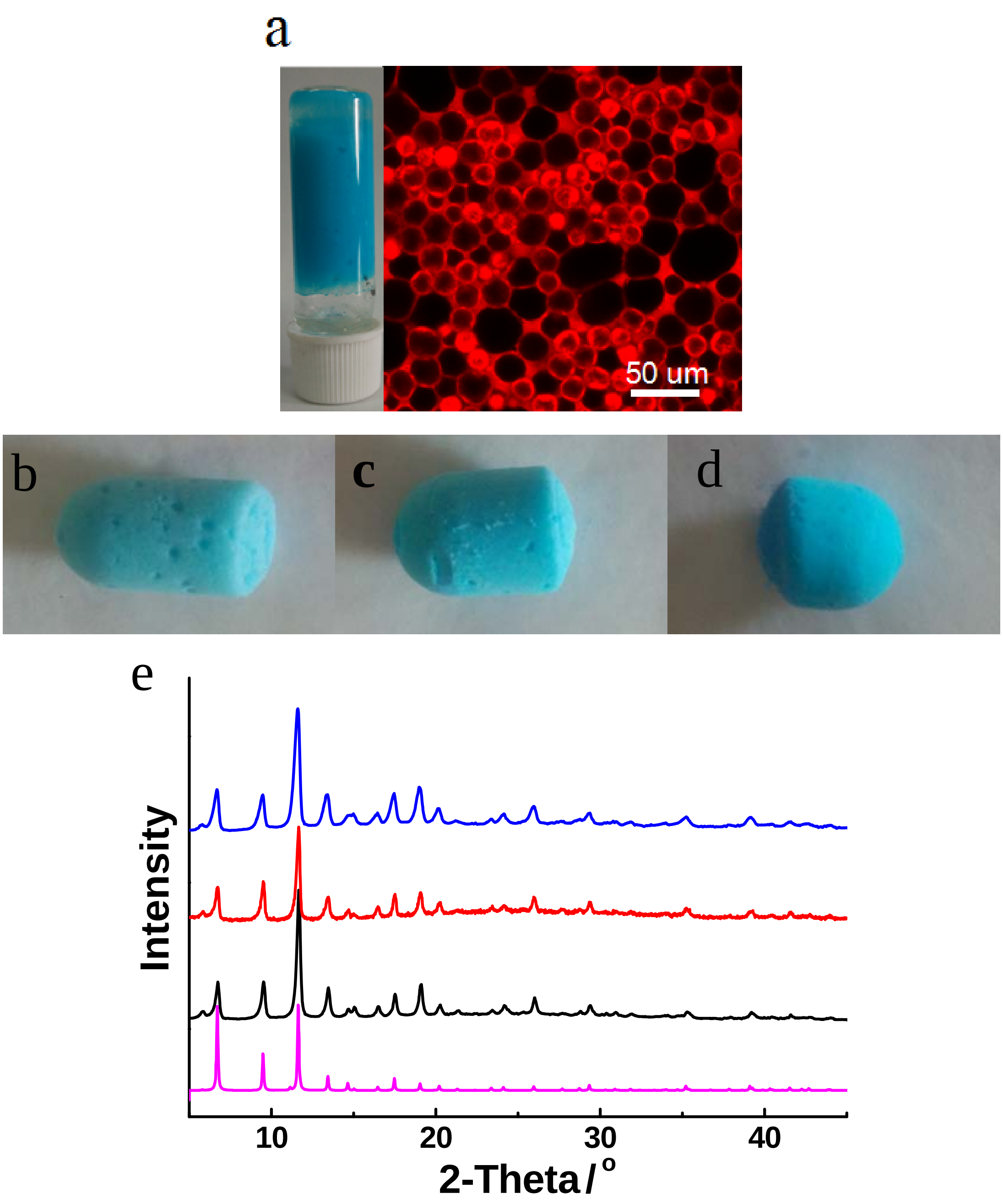

Figure 7. a: Photograph of the emulsion stabilized by $Cu_3(BTC)_2$ with the initial cyclohexane volume fraction of 0.57 and the corresponding CLSM image of the HIPE. **b-d:** Photographs of the MOAs synthesized from the cyclohexane-in-water HIPEs with the cyclohexane volume fractions of 0.57, 0.43, and 0.29, respectively. **e:** Simulated XRD pattern of $Cu_3(BTC)_2$ (pink), XRD patterns of the MOAs synthesized from the cyclohexane-in-water HIPEs with the cyclohexane volume fractions of 0.57 (black), 0.43 (red), and 0.29 (blue), respectively.

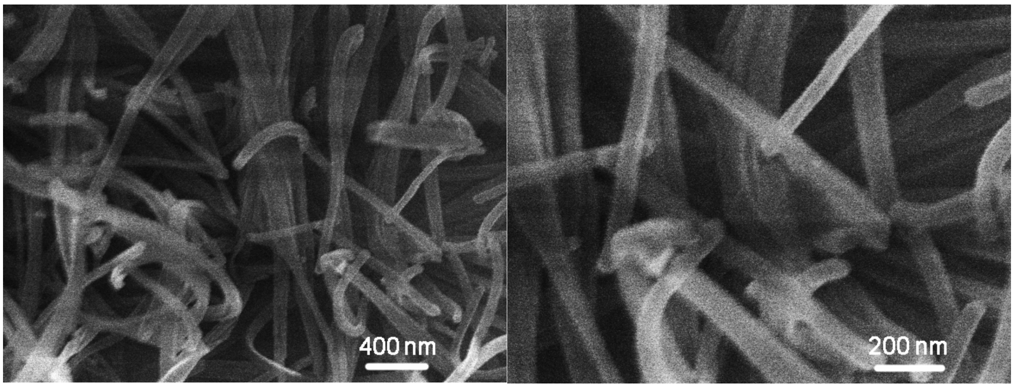

Figure 8. SEM images of the pristine $Mn_3(BTC)_2$ MOF.

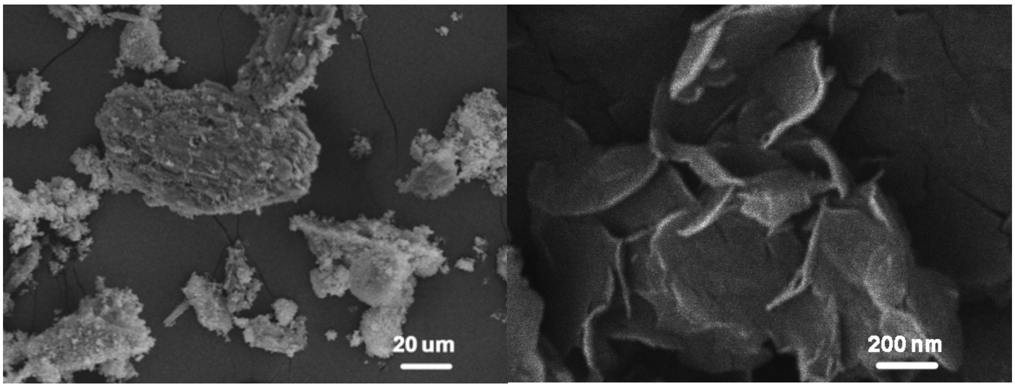

Figure 9. SEM images of the pristine Ni(BDC) MOF.

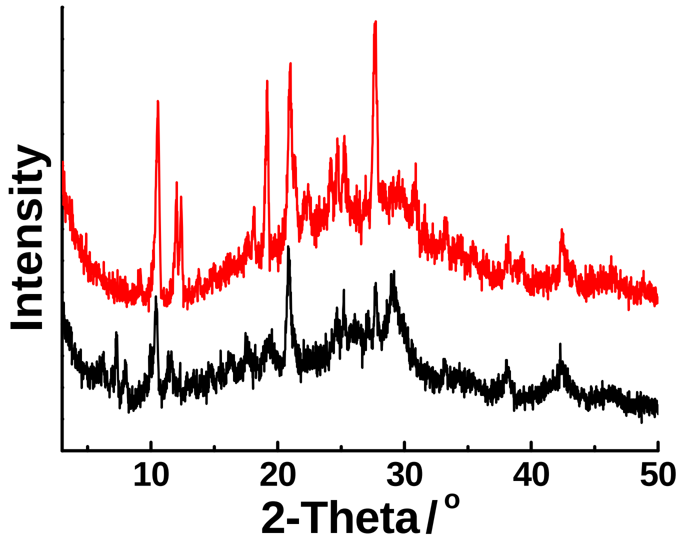

Figure 10. XRD patterns of the pristine $Mn_3(BTC)_2$ MOF (black) and the Mn-MOA (red).

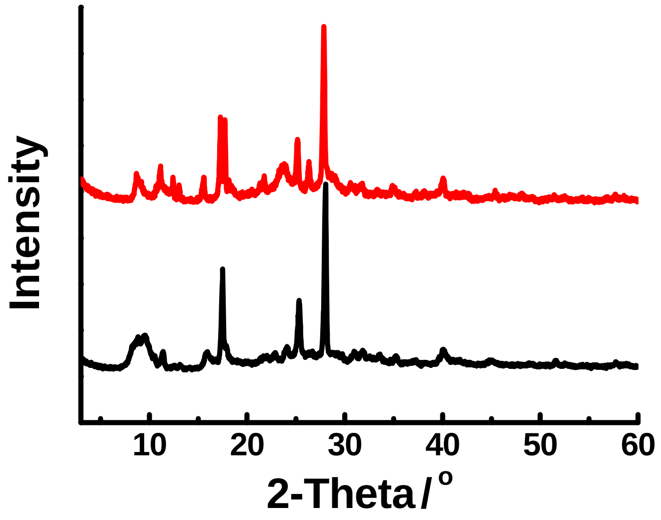

Figure 11. XRD patterns of the pristine Ni(BDC) MOF (black) and Ni-MOA (red).
